# Sparse RNA folding revisited: space-efficient minimum free energy structure prediction

**DOI:** 10.1186/s13015-016-0071-y

**Published:** 2016-04-23

**Authors:** Sebastian Will, Hosna Jabbari

**Affiliations:** Bioinformatics/IZBI, University Leipzig, Härtelstrasse 16–18, Leipzig, Germany; Ingenuity Lab, 11421 Saskatchewan Drive NW, Edmonton, Canada; National Institute for Nanotechnology, 11421 Saskatchewan Drive NW, Edmonton, Canada; Department of Chemical and Materials Engineering, University of Alberta, Edmonton, Canada

**Keywords:** Space efficient sparsification, Pseudoknot-free RNA folding, RNA secondary structure prediction

## Abstract

**Background:**

RNA secondary structure prediction by energy minimization is *the* central computational tool for the analysis of structural non-coding RNAs and their interactions. Sparsification has been successfully applied to improve the time efficiency of various structure prediction algorithms while guaranteeing the same result; however, for many such folding problems, space efficiency is of even greater concern, particularly for long RNA sequences. So far, space-efficient sparsified RNA folding *with fold reconstruction* was solved only for simple base-pair-based pseudo-energy models.

**Results:**

Here, we revisit the problem of space-efficient free energy minimization. Whereas the space-efficient minimization of the free energy has been sketched before, the reconstruction of the optimum structure has not even been discussed. We show that this reconstruction is not possible in trivial extension of the method for simple energy models. Then, we present the time- and space-efficient sparsified free energy minimization algorithm SparseMFEFold that guarantees MFE structure prediction. In particular, this novel algorithm provides efficient fold reconstruction based on dynamically garbage-collected trace arrows. The complexity of our algorithm depends on two parameters, the number of candidates *Z* and the number of trace arrows *T*; both are bounded by $n^2$, but are typically much smaller. The time complexity of RNA folding is reduced from $O(n^3)$ to $O(n^2+nZ)$; the space complexity, from $O(n^2)$ to $O(n + T + Z)$. Our empirical results show more than 80 % space savings over RNAfold [Vienna RNA package] on the long RNAs from the RNA STRAND database (≥2500 bases).

**Conclusions:**

The presented technique is intentionally generalizable to complex prediction algorithms; due to their high space demands, algorithms like pseudoknot prediction and RNA–RNA-interaction prediction are expected to profit even stronger than “standard” MFE folding. SparseMFEFold is free software, available at http://www.bioinf.uni-leipzig.de/~will/Software/SparseMFEFold.

## Background

The manifold catalytic and regulatory functions of non-coding RNAs are mediated by the formation of inter-molecular structures with other RNAs or proteins, as well as their intra-molecular structures [3, 5, 9]. Currently computational RNA structure prediction methods mainly focus on predicting RNA secondary structure—the set of base pairs that form when RNA molecules fold. There is evidence that RNA molecules in their natural environments tend to fold into their minimum free energy (MFE) secondary structure [14]. This motivates various algorithms that predict MFE secondary structures of RNAs. Commonly, the free energy of a secondary structure is calculated by summing up the energies of its single features, where these energies are empirically determined [8]. MFE prediction is applicable in cases of novel RNAs with unknown function, design applications in biotechnology and interacting RNAs.

Recently, sparsification techniques were applied to improve time and space efficiency of various RNA folding algorithms, while guaranteeing the same result. Wexler et al. [15] reduced the time complexity of standard MFE RNA folding by saving redundant recursion cases in the complexity-limiting step of the dynamic programming (DP) algorithm. For this purpose, they introduced candidates, which—by and large—are understood as sub-instances that cannot be optimally partitioned into two smaller sub-instances (confer the simple folding recursions of Fig. 2).

The approach of Wexler et al. which solely improves time efficiency, was implemented for the full free energy model by Dimitrieva and Bucher [4]. Beyond standard folding, sparsification has been studied for more complex folding algorithms, namely pseudoknot folding [10] and RNA–RNA-interaction [13].

Backofen et al. [2] showed that the concept of candidates can be extended to improve time *and* space of RNA folding in base-pair-based (bp-based) pseudo-energy models (i.e. a generalized form of base pair maximization [11]). The two subproblems, energy minimization and fold reconstruction, are commonly solved by DP and trace-back through the DP matrix, respectively. Instead of storing the entire DP matrix, Backofen et al. [2] saved space by storing only a single matrix row (in the case of MFE prediction, several rows) as well as a list of candidates. For bp-based models, this suffices to solve the energy minimization subproblem, and at the same time allows efficient reconstruction of the optimal structure by recomputing matrix rows during trace-back. Note that [13] transferred Backofen et al.’s space savings to MFE RNA–RNA-interaction prediction, however only without space-efficient fold reconstruction.

*Contributions* We show that the fold reconstruction method suggested by Backofen et al. cannot trivially be transferred beyond bp-based models. Consequently, we present a space-saving sparse MFE prediction algorithm with fold reconstruction. In preparation, we revisit space-efficient MFE folding without fold reconstruction. We describe this algorithm including multiloop penalties, i.e. in the variant of Zuker and Sankoff [17], because multiloop penalties are essential for accurate folding and therefore implemented by modern RNA folding software [7]; to the best of our knowledge, the sparsification of MFE prediction with multiloop penalties is elaborated here for the first time. Our efficient fold reconstruction algorithm keeps the additionally required memory to a minimum due to garbage collection. Whereas we describe our techniques for the most common case of RNA MFE folding, they are intentionally more general; in particular, they can be transferred to complex sparsified folding algorithms (e.g., [10, 13]), as well as simultaneous alignment and folding, which profit from sparsification even stronger than standard folding.

## Methods

### Preliminaries of RNA secondary structure prediction

**Fig. 1:**
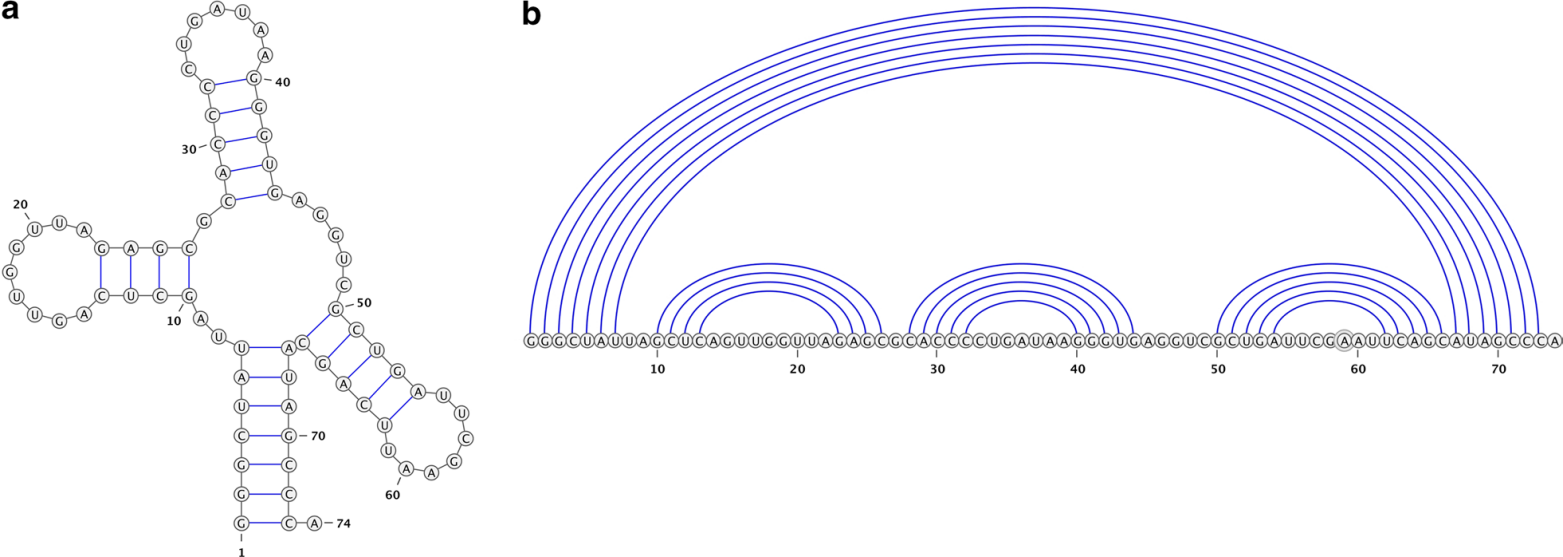
Example of a pseudoknot-free secondary structure **a** in 2D-graphical layout and **b** as* linear arc* diagram. Both representations show the backbone connections of bases in sequence and connect pairing bases

An *RNA sequence*$S=S_1,\dots ,S_n$ is represented as a sequence over the alphabet $\{A,C,G,U\}$. $S_{i,j}$ denotes the subsequence $S_i,\dots ,S_j$. We refer to $S_{i,j}$ as *region* [*i*, *j*]. Fix an RNA sequence *S* of length *n*. A base pair of *S* is an ordered pair *i*.*j* with $1 \le i < j \le n$, such that *i*th and *j*th bases of *S* are complementary (i.e. $\{S_i,S_j\}$ is one of $\{A,U\}, \{C,G\},$ or $\{G,U\}$). A secondary structure *R* for *S* is a set of base pairs such that for all *i*.*j*, $i'.j'\in R$: $\{i,j\}\cap \{i',j'\}=\emptyset$. The base pairs of the secondary structure *R* partition the unpaired bases of sequence *S* into loops [12] (i.e., hairpin loops, internal loops and multiloops). Hairpin loops have a minimum length of *m*; consequently, $j-i>m$ for all base pairs *i*.*j* of *R*. A secondary structure *R* is pseudoknot-free if it does not contain *i*.*j* and $i'.j'$ such that $i<i'<j<j'$. Figure 1 provides an example drawings of a pseudoknot-free secondary structure in 2D layout (Fig. 1a) and the corresponding linear arc diagram (Fig. 1b).

### Energy models for RNA structure prediction

Computational RNA structure prediction minimizes the energy over the large pool of all possible structures, where the energy of a structure is defined by a specific energy model. Certain additive energy models allow efficient optimization by DP—as long as the complexity of possible (i.e., finite energy) structures is limited, e.g. by ruling out pseudoknots.

The simplest energy models are base-pair-based (bp-based); they assign pseudo-energies to base pairs and define the energy *E* of a structure *R* as the sum of energies $E^{\mathrm {bp}}(i.j)$ over all base pairs $i.j\in R$. In the most prominent example of a bp-based model, prediction simply looks for maximizing the number of base pairs. In terms of energy, this is equivalent to the model that assigns $-1$ to each complementary base pair and forbids non-complementary ones.

Biologically relevant RNA structure prediction requires loop-based energy models, where the energy of a secondary structure *R* is defined as sum of the loop energies, i.e. $E(R) = \sum _{\ell \in {\text {loops}}(R)} E^{\mathrm {loop}}(\ell ).$ We denote the free energy of the hairpin loop closed by *i*.*j* by $\mathcal {H}(i,j)$; the energy of internal loops (subsuming stacked and bulge loops) closed by *i*.*j* with inner base pair *k*.*l* is $\mathcal I(i,j, k,l)$; and the free energy of multiloops is calculated from their numbers of inner base pairs *p* and unpaired bases *q* as $\mathcal {ML}(i,j, p,q) = a+b\,p+c\,q$ [8]. Commonly, the size of internal loops is limited to $M$, which caps the time complexity to $O(n^3)$.

Since bp-based models are insufficient for realistic structure prediction—most crucially, they cannot capture stacked loops,—we reserve the term *free energy minimization* for optimization in loop-based energy models. Nevertheless, we start our algorithmic expositions by reviewing the sparsification of bp-based prediction, which is fundamentally simpler than the loop-based case.

### Time and space efficient bp-based folding

The minimum bp-based energy of structures is efficiently computed by DP. Generally, matrix cells with indices (*i*, *j*), referred to as *L*(*i*, *j*), contain minimum bp-based energies for the region [*i*, *j*]. Entries *L*(*i*, *j*) contain the minimum over *all* possible structures, such that the final minimum energy is computed in *L*(1, *n*). $L^c(i,j)$ is the minimum energy of the *closed* substructures of [*i*, *j*] where *i*.*j* is a base pair, and $L^p(i,j)$ minimizes over the *partitionable* substructures of [*i*, *j*], which can be partitioned into two substructures of regions $[i, k-1]$ and [*k*, *j*].

**Fig. 2:**
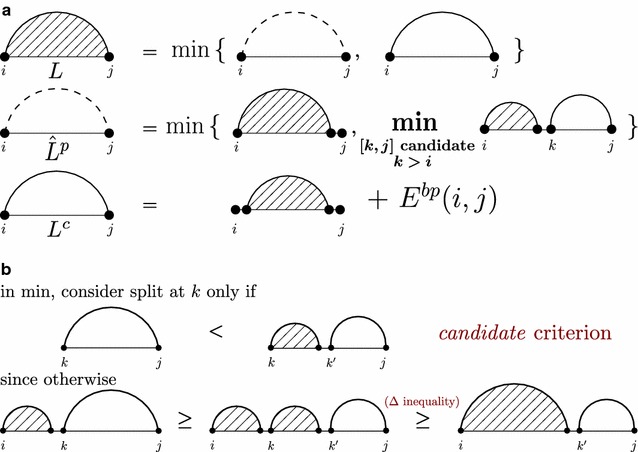
**a** Graphical representation of the sparse bp-based energy minimization recursions. A minimum energy general substructure (*L* *lined pattern*) over region [*i*, *j*] is a closed structure ($L^c$ *solid arcs*) or it is partitionable into two substructures ($\hat{L}^p$ *dotted arcs*). Sparsification restricts the minimization over the partitions in the second row to consider only candidates ${[k,j]}$ for the second fragment. **b** Justification of the *candidate criterion* for sparse bp-based energy minimization according to the recursion of subfigure **a** Candidates are defined as regions [*i*, *j*] where $L^c(i,j)<\hat{L}^p(i,j)$

$$\begin{aligned} L(i,j) &= \min \left\{ \begin{array}{l} L^p(i,j),\\ L^c(i,j) \end{array}\right. \\ L^p(i,j) &= \min \left\{ \begin{array}{l} L(i,j-1),\\ \min \nolimits _{i<k<j} L(i,k-1) + L(k,j) \end{array}\right. \\ L^c(i,j) &= L(i+1,j-1) + E^{\mathrm {bp}}(i.j) \end{aligned}$$

where $$1\le i<j\le n$$, $$L^p(i,i)=L^c(i,i)=+\infty$$ and $$L(i,i)=0$$.

We obtain equivalent sparsified recursions after *replacing*$L^p(i,j)$ by $\hat{L}^p(i,j)$^1^:

$$\begin{aligned} \hat{L}^p(i,j) &= \min \left\{ \begin{array}{l} L(i,j-1), \\ \min \nolimits _{{[k,j]} \,\text {is candidate,} \,k>i} L(i,k-1) + L^c(k,j) \end{array}\right. \qquad ({\hat{L}^p}) \end{aligned}$$

where ${[i,j]}$ is an *L-candidate*, i.e., a candidate for recursion *L*, iff $L^c(i,j)<\hat{L}^p(i,j)$ (see Fig. 2). If, for $i<j$, ${[i,j]}$ is not an *L*-candidate, we call it *L-partitionable*. Note that here we consider ${[i,i]}$ as neither candidates nor partitionable, whereas in [2] they are considered as candidates. To prove the correctness one has to show $\hat{L}^p(i,j)=L^p(i,j)$; this follows the *triangle inequality*$L(i,j)\le L(i,k-1)+L(k,j)$ (for all $1\le i<k\le j\le n$) [2]. Figure 2b depicts the correctness of candidate criterion.

Backofen et al. [2] improved the time and space efficiency of $O(n^3)$ and $O(n^2)$ in the non-sparsified version to $O(n^2 + n\cdot Z_L)$ and $\Theta (n + Z_L)$ respectively, where $Z_L$ is the total number of candidates; typically $Z_L<\!< n^2$. The efficient implementation, which computes the matrix entries row by row starting with row *n*, is based on two further observations: (1) During the DP algorithm, one can maintain an appropriate data structure that allows traversing the candidates ${[k,j]}$ of Eq. (ˆLp) in time linear to the number of candidates ${[k,j]}$. The data structure takes $\Theta (Z_L)$ space. (2) In addition to storing $L^c$ for all candidates in $\Theta (Z_L)$ space, for computing row *i*, it suffices to store the rows *i* and $i+1$, the latter for accessing $L(i+1,j-1)$, at any given time in the DP evaluation.

**Fig. 3:**
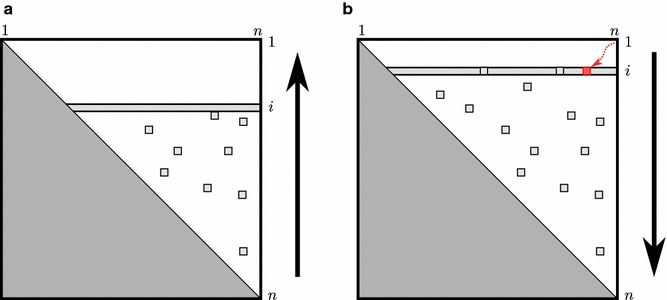
Illustration of bp-based folding and traceback. **a** forward evaluation of the recursions by DP. **b** fold reconstruction by traceback

Figure 3a illustrates the dynamic programming algorithm for minimizing the bp-based energy. To compute *L*(1, *n*), only matrix cells where $i<j$ need to be computed (white region of matrix in Fig. 3a). These cells are filled in bottom-up order, where the calculation of values in row *i* requires only values of row $i+1$ and stored candidates (light-grey boxes). When *L*(1, *n*) is calculated, the *fold reconstruction* is performed by trace-back; this is illustrated in Fig. 3b. For any entry (red box) the continuation of the traceback can be inferred after recomputing the corresponding row *i*; this does not require access to any row greater than *i*. We will revisit this procedure and a more generalized case of fold reconstruction in "The difficulty of MFE fold reconstruction compared to bp-based folding" section.

### Preliminaries on loop-based RNA folding

Before we explain the time and space efficient calculation of the MFE structure, we briefly review the different recurrences of standard loop-based MFE RNA folding.

Efficient MFE structure prediction relies on two pivotal properties: (1) a pseudoknot-free structure over region [*i*, *j*] is either closed by *i*.*j*, or is partitionable at base *k*, $i<k \le j$, into two independent substructures over regions $[i,k-1]$ and [*k*, *j*]; (2) in loop-based energy models, the total energy of a structure composed of two independent substructures is the sum of the energies of its substructures. Therefore a DP algorithm can efficiently minimize the free energy over the pseudoknot-free secondary structures of a given RNA molecule [16, 17].

The free energy minimization algorithm of Zuker and Sankoff [17] calculates the MFE over all pseudoknot-free secondary structures for all subsequences $S_i \ldots S_j$ in matrix cells $W(i,j).$

$$\begin{aligned} W(i,j) = \min \left\{ \begin{array}{l} V(i,j) ,\\ \min \nolimits _{i<k \le j} W(i,k-1) + W(k,j) \end{array}\right. \end{aligned}$$

where $i<j$, and $W(i,i)=0$. Here $V(i,j)$ is the free energy of the pseudoknot-free structure over the region [*i*, *j*] closed by *i*.*j*, and is expressed as follows.where $i<j-m$ (recall that *m* is the minimum size of a hairpin loop), and $V(i,j)=+\infty$ otherwise. We bound the loop size to $M{}$ to keep the time complexity within $O(n^3)$. $V\!M(i,j)$ is the energy of a multiloop over region [*i*, *j*] closed by *i*.*j*.

$$\begin{aligned} V\!M(i,j) = \min \nolimits _{i+1<k \le j-1}(W\!M(i+1,k-1)+W\!M(k,j-1) + a + b) \end{aligned}$$

where *a* is the penalty for multiloop initiation and *b* penalizes inner base pairs in multiloops (also referred to as branch penalty). $W\!M(i,j)$ is the MFE of non-empty structures over region [*i*, *j*] that are inside a multiloop.

$$\begin{aligned} W\!M\,(i,j) = \min \left\{ \begin{array}{l} V(i,j)+b, \\ W\!M(i+1,j)+c, W\!M(i,j-1)+c ,\\ \min \nolimits _{i<k \le j} W\!M(i,k-1) + W\!M(k,j) \end{array}\right. \end{aligned}$$

where $i<j$, and otherwise $W\!M(i,j)=+\infty$. *c* is the penalty for an unpaired base in a multiloop.

## Results

### Time and space efficient calculation of the MFE

In preparation for sparsification, we introduce the matrices $W^p$ and $W\!M^2$, which are not part of the original equations. $W^p(i,j)$ is the energy of the MFE structure in which the MFE structure can be decomposed into two independent subparts. $W\!M^2$ represents multi-loop fragments with at least two inner base pairs. The term $W\!M^2(i+1,j-1)+a +b$ corresponds to the energy of a MFE structure for $S_i \ldots S_j$ in which *i*.*j* closes a multiloop; in the previous section, this is denoted by $V\!M(i,j)$. $W\!M^p(i,j)$ is the energy of the MFE $W\!M(i,j)$ structure in which the structure can be partitioned into two independent subparts.where $i<j$, $W(i,i)=0$; $V(i,j)=W\!M(i,j)=\infty$ for all $j-i\le m$; and $W\!M^2=\infty$ for all $j-i\le 2m+3$.

We sparsify the recurrences by rewriting $W^p(i,j)$ to $\widehat{W}^\mathrm{p}(i,j)$ and $W\!M^2(i,j)$ to $\widehat{W\!M}{^2}(i,j)$, where

$$\begin{aligned}\widehat{W}^\mathrm{p}(i,j) &= \min \left\{ \begin{array}{l} W(i,j-1), \\ \min \nolimits _{[k,\, j]\text{ is W-candidate},\, k>i} W(i,k-1) + V(k,\, j) \end{array}\right. \\ \widehat{W\!M}{^2}(i,\,j) &=\min \left\{ \begin{array}{l} W\!M^2(i,j-1) + c, \\ \min \nolimits _{[k,\, j]\text{ is WM-candidate},\, k>i} W\!M(i,k-1) + V(k,\, j) \end{array}\right. \end{aligned}$$

together with the candidate criteria

- ${[k,j]}$ is a *W-candidate* iff $V(k,j) < \widehat{W}^\mathrm{p}(k,j)$ and
- ${[k,j]}$ is a *WM-candidate* iff $V(k,j)+b < W\!M^p(k,j)$.

We note that to have similar recurrences, we also rewrite $W\!M^p(i,j)$ recurrence as follows:

$$\begin{aligned} W\!M^p(i,j) = \min \left\{ \begin{array}{l} W\!M(i+1,j) + c, \\ \widehat{W\!M}{^2}(i,j) \end{array}\right. \end{aligned}$$

which merges the second case of original $W\!M^p$ into $\widehat{W\!M}{^2}$ recurrence.

#### **Lemma 1**

*The sparsified version of*$W^p$* and*$W\!M^2$* recurrences are equivalent to the non-sparsified recurrences.*

#### *Proof*

1. Choose the largest *k*, $i < k < j$, s.t. $W(i,k-1) + W(k,j)$ is minimal. We show that [k,j] is W-candidate. Assuming the opposite, choose *e* ($e>k$), such that $W^p(k,j) = W(k,e-1) + W(e,j)$. Now $W^p(i,j) = W(i,k-1) + W(k,e-1) + W(e,j) \ge W(i,e-1) + W(e,j)$, which contradicts the choice of *k* such that $W(i,k-1) + W(k,j)$ is minimal. Therefore we must have $W(k,j) = V(k,j) < \widehat{W}^\mathrm{p}(k,j)$, and ${[i,j]}$ is a *W-candidate*.
2. Choose the largest *k*, $i < k < j$, s.t. $W\!M(i,k-1) + W\!M(k,j)$ is minimal. We show that [k,j] is WM-candidate. Assuming the opposite, choose *e* ($e>k$), such that $W\!M^2(k,j) = W\!M(k,e-1) + W\!M(e,j)$. Now $W\!M^2(i,j) = W\!M(i,k-1) + W\!M(k,e-1) + W\!M(e,j) \ge W\!M(i,e-1) + W\!M(e,j)$, which contradicts the choice of *k* such that $W\!M(i,k-1) + W\!M(k,j)$ is minimal. Therefore we must have $W\!M(k,j) = V(k,j) + b < W\!M^p(k,j)$, and ${[i,j]}$ is a *WM-candidate*.

$$\square$$

Going beyond Wexler et al., these recursions handle multiloop energies correctly by introducing the matrices $W\!M$, $W\!M^p$ and $\widehat{W\!M}{^2}.$

Analogous to [2], there is an algorithm that evaluates the above recursions efficiently, such that time and space complexity depend on $Z$, where $Z$ is the total number of *candidates* (which are *W*- or *WM*-candidates.) We call this algorithm SparseEnergyMinimization.

#### **Lemma 2**

$W(1,n)$* can be calculated in*$O(n^2+nZ)$* time and*$\Theta (n+Z)$* space, where*$Z$* is the total number ofcandidates*.

#### *Proof*

*Time*SparseEnergyMinimization computes $O(n^2)$ entries and performs the minimizations over all candidates in the calculations of $\widehat{W}^\mathrm{p}$ and $\widehat{W\!M}{^2}$. These minimizations require $O(Z)$ steps per matrix row, resulting in $O(nZ)$ additional time.

*Space* To calculate all $$\widehat{W}^\mathrm{p}(i,j)$$ and $$\widehat{W\!M}{^2}(i,j)$$ in row *i* it suffices to compute and store the entries in the same matrix row and store the matrix entries at the candidates of rows $$i'>i$$. For calculating the $$V(i,j)$$ in row *i* ($$j:i<j\le n$$), it suffices to keep row $$i+1$$ of $$WM^p$$ and the rows $$i+1$$ to $$i+M+1$$ of *V* in memory, since the interior loop size is bounded by $$M.$$$$\square$$

### The difficulty of MFE fold reconstruction compared to bp-based folding

The MFE structure in the bp-based model is efficiently reconstructed using the minimum energy, the energies of candidates, and *O* (*n*) space by trace-back with recomputation of partitionable entries, which are not stored in the DP-matrix. We briefly recapitulate this result of [2].

#### **Lemma 3**

*The optimal structure in the bp-based model can be reconstructed from the candidates and the minimum free energy in*$O(n+Z_L)$* space and*$O(n^2+nZ_L)$* time.*

#### *Proofsketch*

The algorithm starts similar to a regular trace-back from *L*(1, *n*). Recursively, it derives the optimum recursion cases of the current matrix entry and continues to trace back from the identified successive trace entries. For finding the successive trace entries from a current entry (*i*, *j*), it suffices to know the entries $$(i,j')$$ ($$j'\le j$$) of the same row: if $${[i,j]}$$ is a candidate, then the successive trace entry is $$(i+1,j-1)$$; otherwise, it can be split at some *k*, s.t. entry $$(i,k-1)$$ is in the same row and $${[k,j]}$$ is a candidate (unless $$k=j$$). On demand, the entries $$(i,j')$$ can be recomputed from entries $$(i,j'')$$$$(j''<j')$$ of this row and candidate entries. Note that access to non-candidates of rows $$i'>i$$ is never required. In particular, the algorithm utilizes that the candidates $${[i,j]}$$ of row *i* do not have to be recomputed, because candidates necessarily trace back to $$(i+1,j-1)$$. Thus, the trace-back with recomputation takes $$O(n\cdot Z_L)$$ time and does not require additional space. $$\square$$

After executing SparseEnergyMinimization, all candidates are calculated and stored in memory, analogously to the bp-based case. However, there is no trivial transfer of the bp-based trace-back algorithm of [2], Folding-Traceback, to the loop-based case.

**Fig. 4:**
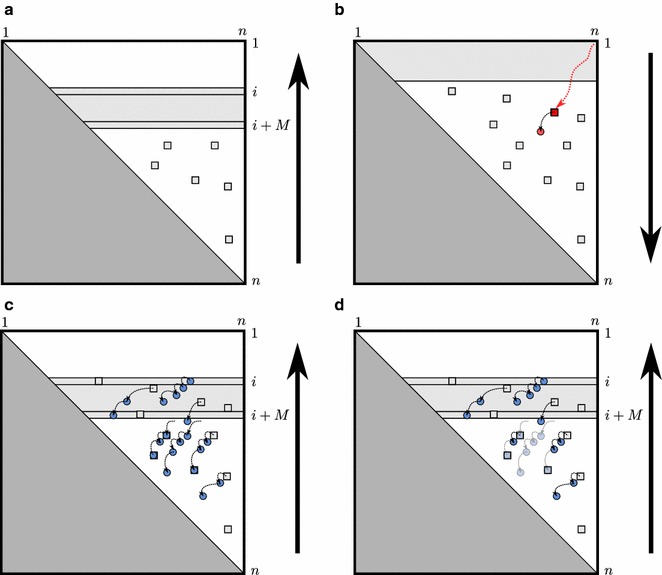
Ideas of the space-efficient backtracking procedure and its requirements. We illustrate concepts sketching the upper-triangular *V*-matrix in different algorithm phases. The* dark-grey lower triangle* is unused. The stored rows *i*..$i+M$ are shown as* light-grey* area; the* light-grey boxes* represent the candidates. **a** Evaluation phase of sparse MFE folding without space-efficient trace-back; the algorithm stores the last $M+1$ rows and the candidates. **b** Space-efficient trace-back (*red arrow*) in the basic algorithm fails, since trace continuations cannot be efficiently retrieved in general; eventually, the trace reaches a non-candidate entry (*filled circle*), which is not in memory. **c** Naive sparse MFE folding with trace-back stores all trace arrows (*black arrows*) to candidates (*boxes*) and non-candidates (*circles*). **d** SparseMFEFold removes* arrows* to candidates and applies garbage collection to fundamentally reduce the space requirements

The main difference between the bp-based and the loop-based folding algorithm is the evaluation of interior loops. In both cases, bp-based folding and loop-based folding, the energy of a closed structure, respectively $L^c(i,j)$ and *V*(*i*, *j*), depends only on a constant number of rows (resp., 1 row like in Fig. 3a or $M{}$ rows like in Fig. 4a.) However, Folding-Traceback relies on the fact that the successive trace entry of candidates is known, whereas the MFE fold reconstruction has to infer the optimum recursion case of *V*(*i*, *j*), even if $V(i,j)<\min \{W(i,j),W\!M(i,j)\}$—corresponding to the optimum co-terminus criterion of [2]. Thus, the efficient reconstruction of the MFE fold is not directly possible from the candidates alone, since in general the traceback requires the MFE of non-candidate regions. This is illustrated in Fig. 4b, where continuing beyond the red trace-back arrow requires an unavailable matrix cell, which was not stored.

Naively, this requires to recompute the (non-candidate) $V$ entries of rows $i+1 \, ,\ldots, \, i+M+1$, which in turn rely on $V$ and $W\!M$ entries of larger rows. Consequently, the non-candidate entries of the whole $V$ matrix have to be recomputed. This negates the sparsification benefits. Furthermore, there seems to be no simple way to overcome this problem. In particular, we cannot directly compute *V*(*i*, *j*) by minimizing only over candidates, since there is no guarantee that the inner base pair of an interior loop corresponds to a candidate.

#### **Lemma 4**

*The minimization over inner base pairs in the recursion of *$V$* cannot be restricted to candidates.*

#### *Proof*

We show that there is a loop-based energy model (namely the Turner energy model [8]), a sequence *S* and $1\le i<j\le n$, such that $V(i,j) < \min \{ \mathcal {H}(i,j), W\!M^2(i+1,j-1)+a \},$ but there is no candidate ${[p,q]}$, $i<p<q<j$, such that $V(i,j)=\mathcal I(i,j,p,q) + V(p,q).$

Consider the RNA sequence $S=\texttt {GCCAAAAGGGC}$ of length 11. In the Turner model, the optimal recursion case of $V(2,10)$ forms the interior loop closed by (2, 10) with inner base pair (3, 9), because $V(3,9)=\mathcal {H}(3,9)=4.3$ kcal/mol and $\mathcal I(2,10,3,9)=-3.3$ kcal/mol. However, ${[3,9]}$ is not a candidate, since $W(3,9)=W\!M(3,9)=\mathcal {H}(3,8)=4.1 < V(3,9),$ i.e. the MFE structure of $S_{3,9}$ forms the hairpin loop closed by (3, 8)—not by (3, 9). $\square$

The lemma holds for arbitrarily large instances. This can be seen by, for example, looking at the family of RNA sequences $S_{k}=GC_k A_4 G_{k+1} C$, where $X_k$ is the *k*-times repetition of *X*. Furthermore, this issue is not limited to stacked base pairs, since there are non-stacked interior loops with stabilizing energy contributions, in the Turner energy model.

### Overview of MFE folding with fold reconstruction

As discussed earlier direct transfer of Folding-Traceback from [2] is not possible because the optimum case of the $V$-recursion cannot be determined efficiently by recomputation. Therefore we suggest to store trace arrows from all entries that cannot be recomputed efficiently. Subsequently, we discuss several space optimizations for this idea, such as avoiding trace arrows by rewriting the recursions and removing trace arrows as soon as they become inaccessible for the trace-back.

### Adding trace arrows

As a first step towards efficient trace-back, we store trace arrows from each potential base pair *i*.*j* to its optimum inner base pair during the DP evaluation. Here, a *trace arrow* is simply a directed edge connecting two matrix entries. By storing these arrows we avoid the recomputation of all $V$ entries in the trace-back, by inferring their successive trace entries. If there is no trace arrow to an inner base pair and $V(i,j)\ne \mathcal {H}(i,j)$, we can simply continue to trace from $(i+1,j-1)$ in matrix $W\!M^2.$

Furthermore, the case $W\!M(i+1,j) + c$ of $W\!M^p$ accesses entries beyond the current row *i*. As before, we cannot efficiently recompute row $i+1$, which could be resolved by recording trace arrows. Figure 4c depicts the naïve ad-hoc solution to the fold reconstruction problem by adding all trace arrows.

### Avoiding trace arrows

One can avoid the trace arrows for the case $W\!M(i+1,j) + c$ of $W\!M^p$ by rewriting the case equivalently as follows:

$$\begin{aligned} W\!M^p(i,j) &= \min \left\{ \begin{array}{l} W\!M(i+1,j) + c, \\ W\!M^2(i,j) \end{array}\right. \\ &= \min \left\{ \begin{array}{l} \min \nolimits _{i<k<j} (k-i) \times c + W\!M(k,j),\\ W\!M^2(i,j) \end{array}\right. \end{aligned}$$

Since $W\!M(i,i) = +\infty$, we can sparsify the recurrence as follows:We have already shown the equivalence of $\widehat{W\!M}{^2}$ and $W\!M^2$ recurrences; thus, we establish the correctness of this rewriting by the following lemma. This serves well as an example of a typical *small* change during sparsification of recursions, which is nevertheless non-trivial.

#### **Lemma 5**

*Replacing*$W\!M^p$* by*$\widehat{W\!M}^\text {p}$* leaves the values of *$W$, $V$,* and*$W\!M$* entries unchanged.*

#### *Proof*

We have to show that restricting the minimization $$\min \nolimits _{\begin{array}{c} i<k<j \end{array}} (k-i) \times c + V(k,j) + b$$ to only $W\!M$-candidates is admissible; this boils down to showing that non-candidates in the new minimization do not change the minimum values in the recursions. Assume that ${[k,j]}$ is $W\!M$-partitionable. By definition there exists a $k'>k$, where ${[k',j]}$ is a $W\!M$-candidate s.t. one of the following holds.

1. $$V(k,j)+b \ge (k'-k) \times c + V(k',j) + b$$
2. $$V(k,j)+b \ge W\!M(k,k'-1) + V(k',j) + b$$

**Case 1.**$(k-i)\times c + V(k,j) + b \ge (k-i)\times c + (k'-k)\times c + V(k',j) + b \ge (k'-i)\times c + V(k',j) + b$, i.e. $k'$ dominates *k* in this minimization.

**Case 2.**$(k-i)\times c + W\!M(k,k'-1) + V(k',j) + b \ge W\!M(i,k'-1) + V(k',j) + b$; since the latter is a case of $\widehat{W\!M}{^2}$, *k* is again dominated. $\square$

Furthermore, we do not have to store trace arrows from $V$ entries (*i*, *j*) to candidates. In such cases, the optimum interior loop case, $V^{\text {il-cand}}(i,j)$, can be reconstructed by minimizing over all candidates:1If $V^{\text {il-cand}}(i,j)$ is the MFE, we have reconstructed the trace arrow, however there is one catch: recall that we cannot use $W\!M^2(i+1,j-1)+a+b$ to decide whether $W\!M^2(i+1,j-1)+a +b < min\{\,V^{\text {il-cand}}(i,j),\mathcal {H}(i,j)\,\}$, because it is neither stored nor can be recomputed efficiently. Thus, our strategy is to trace into the multiloop, iff no other case yields the MFE $V(i,j)$. However, as of this computation, we do not know this energy for non-candidate entries during trace-back. Therefore, we additionally keep track of this energy: each time we trace back from some (*i*, *j*) to a non-candidate (*p*, *q*), we recalculate the entry $V(p,q)$ due to $V(p,q) = V(i,j) - \mathcal I(i,j,p,q).$

### Garbage collecting trace arrows

So far, our algorithm stores all trace arrows from $V$ entries to non-candidates. However, most of those $V$ entries are not on the MFE trace (rather far off from it). Identifying unnecessary arrows during the recursion evaluation, allows saving space for trace arrows, while still supporting MFE fold reconstruction.

Of course, during the evaluation we generally have only partial information about the MFE trace. Therefore, a safe strategy is to remove the trace arrows that are inaccessible from current and future accessible entries.

We define a directed graph $G=(\mathcal {V},\mathcal {E})$, in which cells of the DP matrix are represented as vertices and there is a directed edge between two vertices $\mathcal {V}_1$ and $\mathcal {V}_2$ if there is a trace arrow from $\mathcal {V}_1$ to $\mathcal {V}_2$.

#### **Definition 1**

An entry *V*(*p*, *q*) is accessible, iff during recursion evaluation, after computing row *i*:

- $$p \le i+M+1$$,
- ${[p,q]}$ is a candidate, or
- there is a trace arrow from some accessible entry to *V*(*p*, *q*).

Detecting inaccessible entries in such a directed graph structure can be performed by garbage collection (GC) [6]. Since there is no cycle in our directed graph, we apply a simple reference counting GC technique. Each arrow *ta* receives a counter, which keeps track of the arrows that point to the source of *ta*. After computing row *i*, we scan through the arrows with source in row $i+M+1$. Arrows from non-candidates in row $i+M+1$ are removed, if their reference count is zero. In a recursive procedure, we detect all arrows pointing from inaccessible entries, remove them and update the appropriate counters. Figure 4d illustrates avoided trace arrows to candidates and removed inaccessible trace arrows (due to garbage collection).

### Algorithm summary

Employing the above two techniques, our algorithm SparseEnergyMinimization (Algorithm 1) keeps track of trace arrows and performs reference counting garbage collection (Procedure GarbageCollect). Note that for further space savings, the algorithm does not distinguish $W$- and $W\!M$-candidates; this does not affect our complexity bounds.

The final algorithm SparseMFEFold performs energy minimization and fold reconstruction. The fold reconstruction relies on the complete results of Algorithm 1, i.e. the minimum free energy, the candidates, and the trace arrows.
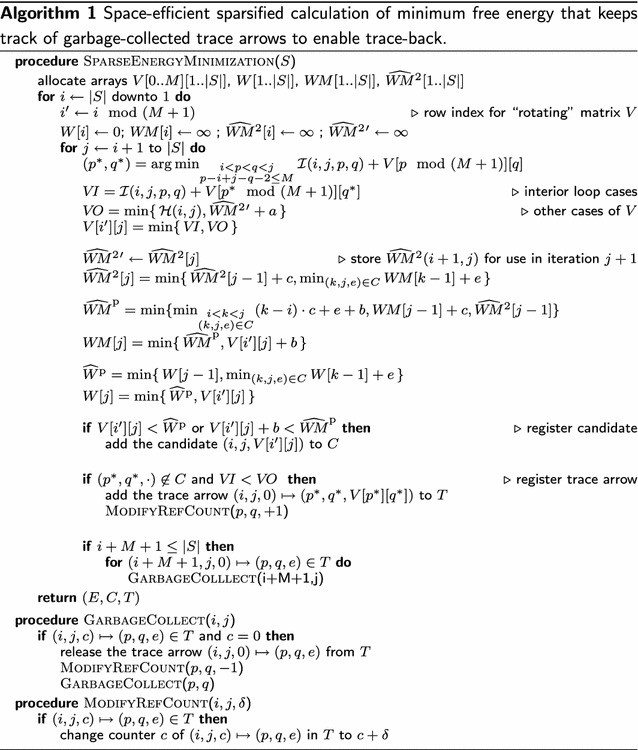


### Complexity

Implementing the trace arrow data structure as a dynamic hash table allows access to a trace arrow by its origin in amortized constant time; such a hash table requires *O*(*T*) space, which is non-critical for the space bound derived below. In the following, we assume constant time access.

#### **Lemma 6**

SparseEnergyMinimization* (including storing and garbage collection of trace arrows) calculates *$W(1,n)$* in*$O(n^2+nZ)$* time and*$\Theta (n+Z+T)$* space, where*$Z$* is the total number ofcandidates* and *T is the maximum number of accessible trace arrows to non-candidates.*

#### *Proof*

The number of trace arrows is quadratically limited and the criterion for storing a trace arrow is checked in constant time, such that the time complexity is not changed. The time for garbage collection of trace arrows is at most quadratic, because GarbageCollect is called at most once per matrix entry from SparseEnergyMinimization. Furthermore, each time it calls itself it removes one trace arrow. Each trace arrow can be inserted and removed only once by SparseEnergyMinimization. The space complexity depends on the maximum number of trace arrows that have to be stored simultaneously. Without garbage collection, this is the number of trace arrows to non-candidates. Due to the garbage collection, we reduce this to the maximum number of simultaneously accessible trace arrows to non-candidates. $\square$

Note that since candidates correspond to *V*-matrix entries, *Z* is bounded by the number of matrix entries in *V*; moreover, *T* is bounded by this numbers since each entry has at most one trace arrow. Thus, SparseMFEFold is guaranteed to stay in the time and space complexities of the original algorithm.

### Empirical results

We implemented the algorithm SparseMFEFold in C++ utilizing the Vienna RNA library [7] for calculating the single loop energies. Consequently, we computed exactly the same energies and structures as RNAfold of the Vienna RNA package 2.x [7] (without dangling ends, i.e., option -d0.) This implementation allows us to study the suggested strategies empirically.

For evaluating the method, we folded all single-molecule RNA sequences from the RNA STRAND v2.0 database [1] with SparseMFEFold and RNAfold from the Vienna RNA package. Moreover, we folded the sequences by SparseMFEFold without GC to assess its performance impact. All experiments were performed on a Lenovo Thinkpad T431s with 12GB memory and Intel i5-3437U CPU. We measured run-time as user time and space consumption as maximum resident set size.

**Fig. 5:**
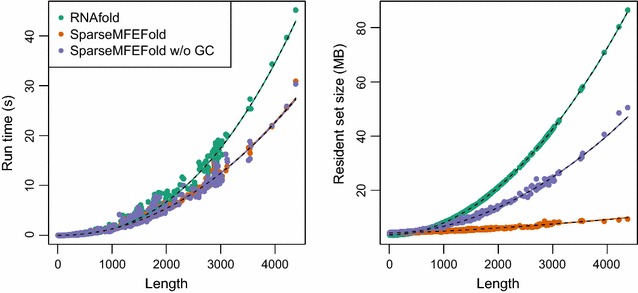
Time and space consumption in dependency of sequence length. The plot illustrates results for all RNA STRAND instances with single molecule folds. We compare RNAfold, SparseMFEFold, and—to show the effect of garbage collection—SparseMFEFold without this feature. The best* fitted curves* are shown as* dashed lines*

*Time and Space Performance Dependence on Sequence Length* Figure 5 compares the performance of our SparseMFEFold algorithm with RNAfold on the RNA STRAND sequences. For the shorter sequences (shorter than 1000 bases), our algorithm’s performance is on-par with RNAfold. However, we observe strong space savings (and slight time improvements) for longer RNAs.

The same plot represents the performance of our algorithm without garbage collection (SparseMFEFold w/o GC). It is evident from Fig. 5 that both versions of SparseMFEFold perform similarly in terms of run time; thus, remarkably, GC does not cause significant time overhead. While already SparseMFEFold w/o GC is superior to RNAfold in terms of space consumption, we see a major improvement due to garbage collection, such that—in comparison—SparseMFEFold’s space consumption increases only minimally over the range of sequence lengths.

We further used a non-linear regression model to find the best fitted curve of the form $o + f \times n^e$ (where *o* is the offset, *f* is the coefficient, *n* is the length of the sequence, and *e* is the exponent) explaining the empirical performance of each algorithm on our data set. Figure 5 represents these curves in dashed lines. We found the best fit for the time of RNAfold to be not cubic but with an exponent of about 2.4; SparseMFEFold has an improved best fitted exponent of about 2.1. We note that in both cases the *f* values are of the same order ($1.01\times 10^{-7}$ vs. $6.9 \times 10^{-7}$, respectively) and the offset values are negligible ($1.0 \times 10^{-2}$ vs. $-3.35 \times 10^{-3}$, respectively). In terms of space, however, the difference of the performance of the two algorithms is more significant; the best fitted curve for RNAfold has the exponent value of 1.98 ($f=5.3 \times 10^{-6}$, and $o=3.6$), while the best exponent for SparseMFEFold has the value of 1.44 ($f=3.1 \times 10^{-5}$, and $o=4.3$).

**Fig. 6:**
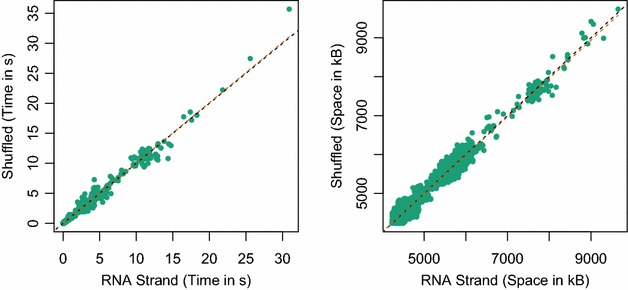
Sparsification of true and shuffled RNAs. We compare the time and space consumption of SparseMFEFold for RNA STRAND instances versus di-nucleotide shuffled RNA STRAND instances. The* dashed lines* show the identity (*black*) and linear fit (*orange*), which are almost indistinguishable

*Sparsification Strength* Remarkably, our results on the entire RNA STRAND sequences show very little variation in sparsification strength. This observation is close to (negatively) answering the long standing question, whether there are RNA classes with particular susceptibility to sparsification. Adding to this observation, we investigated whether sparsification has more significant (or weaker) effect for naturally occurring RNAs compared to random sequences. Thus, we created di-nucleotide shuffle of our pool of benchmark sequences and measured the performance of SparseMFEFold on these random sequence under the same conditions. Figure 6 summarizes the results of this shuffling experiment, reporting time and space. Evidently, no significant difference is observed.

*Detailed Performance Comparison on Long RNAs* Tables 1 and 2 summarize results from folding the 80 longest RNA sequences from the RNA STRAND v2.0 database [1]. This sequence set comprises of all RNAs of length greater or equal 2500, for which a single molecule fold is available. These sequences have a median length of 2904 and a maximum of 4381. Our comparison to RNAfold, currently the fastest RNA folding implementation, shows that our sparsified algorithm is significantly faster and uses significantly less space (Table 1). Note that even the median resident set size of our method is about six-times lower than that of RNAfold. To empirically study the effects of our optimizations in SparseMFEFold, we further provide number of candidates and trace arrows (Table 2). For the trace arrows, we report minimum, median, and maximum of the final number of trace arrows (Final), passed to the fold reconstruction algorithm; the maximum number of trace arrows (Maximum), determining the memory foot print; the savings due to avoiding arrows to candidates (Avoided); and garbage collection of inaccessible arrows (GC-Removed). The latter two numbers show the importance of these two optimizations for the entire approach; together these strategies reduce the (median) number of stored trace arrows to only about $9~\%$ (94,443/1,038,525).

**Table 1:** Time and space performance of SparseMFEFold compared to RNAfold

	Run-time (s)		Memory: resident set size (kB)	
	RNAfold	SparseMFEFold	RNAfold	SparseMFEFold
Minimum	16.9	15.37	31,800	5932
Median	29.7	22.89	42,828	7262
Maximum	89.9	57.36	88,548	9048

**Table 2:** Counts of candidates and trace arrows in SparseMFEFold

	Number of	Number of trace arrows			
	candidates	Final	Maximum	Avoided	GC-removed
Minimum	17,032	49,860	52,293	137,892	467,230
Median	41,215	92,967	94,443	237,717	706,365
Maximum	71,508	147,150	148,947	419,825	1,748,491

## Discussion and conclusions

We identified and solved the fundamental problem of efficient fold reconstruction in time- and space-efficient sparsified MFE folding of RNAs while guaranteeing prediction of the MFE structure. This problem is not present in simple variants of RNA folding such as base pair maximization, but emerges only in realistic free energy minimization problems. Remarkably, Backofen et al. did not mention this problem when discussing the extension of their time and space-efficient base pair maximization algorithms to MFE prediction. Here, we provide an elegant and practical solution, which introduces garbage collection as a novel technique to RNA folding. The method is presented and studied for the most-common case of pseudoknot-free RNA secondary structure prediction using the Turner energy model.

Our algorithm, SparseMFEFold, outperforms RNAfold both in terms of time and space. We note that this performance improvement is specifically more pronounced for longer sequences. Based on our experiments on RNA sequences of RNA STRAND database, we did not notice any difference in performance of our algorithm corresponding to different families of structures. We further compared performance of our algorithm on artificial sequences with the same length as RNA sequences in RNA STRAND database. To create such artificial sequences, we performed di-nucleotide shuffle for each RNA STRAND sequence. By shuffling, we demonstrated that there is no significant difference in the performance of SparseMFEFold (and thus sparsification in general) between naturally occurring RNA sequences and their di-nucleotide shuffled sequences. This suggests that there is no special class of RNAs with either exceptionally strong or weak susceptibility to sparsification; in other words, sparsification is universally advantageous for all classes of RNAs.

Importantly, the introduced techniques are not specific to the presented folding scenario, but are applicable to many—even fundamentally more complex—variants of RNA folding, such as the MFE prediction of RNA–RNA-interactions and efficient pseudoknot folding algorithms. Similar to the case of time-efficient sparsification, the presented techniques will have even stronger impact on complex folding algorithms. Thus, we see the strongest potential of our method in reducing the often prohibitive space requirements of such algorithms.

## Abbreviations

bp-based: base-pair-based
DP: dynamic programming
GC: garbage collection
MFE: minimum free energy
RNA: ribonucleic acid
TA: trace arrow

## Footnotes

1 i.e., we replace the recursion $L^p(i,j)$ by Eq. (ˆLp) and replace the symbol $L^p(i,j)$ by $\hat{L}^p(i,j)$ in the recursion *L*.
